# *C. elegans* EIF-3.K Promotes Programmed Cell Death through CED-3 Caspase

**DOI:** 10.1371/journal.pone.0036584

**Published:** 2012-05-09

**Authors:** Chun-Yi Huang, Jia-Yun Chen, Shu-Chun Wu, Chieh-Hsiang Tan, Ruei-Ying Tzeng, Pei-Ju Lu, Yu-Feng Wu, Ruey-Hwa Chen, Yi-Chun Wu

**Affiliations:** 1 Institute of Molecular and Cellular Biology, National Taiwan University, Taipei, Taiwan; 2 Institute of Molecular Medicine, National Taiwan University, Taipei, Taiwan; 3 Institute of Biochemical Sciences, National Taiwan University, Taipei, Taiwan; 4 Center for Systems Biology, National Taiwan University, Taipei, Taiwan; 5 Research Center for Developmental Biology and Regenerative Medicine, National Taiwan University, Taipei, Taiwan; 6 Institute of Biomedical Sciences, Academia Sinica, Taipei, Taiwan; 7 Institute of Atomic and Molecular Sciences, Academia Sinica, Taipei, Taiwan; NIH-NCI, United States of America

## Abstract

Programmed cell death (apoptosis) is essential for the development and homeostasis of metazoans. The central step in the execution of programmed cell death is the activation of caspases. In *C. elegans*, the core cell death regulators EGL-1(a BH3 domain-containing protein), CED-9 (Bcl-2), and CED-4 (Apaf-1) act in an inhibitory cascade to activate the CED-3 caspase. Here we have identified an additional component *eif-3.K* (eukaryotic translation initiation factor 3 subunit k) that acts upstream of *ced-3* to promote programmed cell death. The loss of *eif-3.K* reduced cell deaths in both somatic and germ cells, whereas the overexpression of *eif-3.K* resulted in a slight but significant increase in cell death. Using a cell-specific promoter, we show that *eif-3.K* promotes cell death in a cell-autonomous manner. In addition, the loss of *eif-3.K* significantly suppressed cell death-induced through the overexpression of *ced-4*, but not *ced-3*, indicating a distinct requirement for *eif-3.K* in apoptosis. Reciprocally, a loss of *ced-3* suppressed cell death induced by the overexpression of *eif-3.K*. These results indicate that *eif-3.K* requires *ced-3* to promote programmed cell death and that *eif-3.K* acts upstream of *ced-3* to promote this process. The EIF-3.K protein is ubiquitously expressed in embryos and larvae and localizes to the cytoplasm. A structure-function analysis revealed that the 61 amino acid long WH domain of EIF-3.K, potentially involved in protein-DNA/RNA interactions, is both necessary and sufficient for the cell death-promoting activity of EIF-3.K. Because human eIF3k was able to partially substitute for *C. elegans eif-3.K* in the promotion of cell death, this WH domain-dependent EIF-3.K-mediated cell death process has potentially been conserved throughout evolution.

## Introduction

Programmed Cell death is an evolutionarily conserved cellular process that eliminates unnecessary, damaged, or harmful cells [1], [2]. Inappropriate regulation of this process can lead to developmental disorders, tumorigenesis, or degenerative pathologies in *C. elegans*, flies, mice, or humans [3].

Molecular and genetic studies in *C. elegans* have led to the identification and characterization of the evolutionarily conserved genes *egl-1*, *ced-3*, *ced-4*, and *ced-9*, which constitute the core cell death pathway [4]–[6]. The proteins encoded by these genes act in an inhibitory cascade. EGL-1(a BH3-containing protein) promotes cell death by antagonizing the cell death inhibitory function of CED-9, a homolog of BCL-2 [5], [7]. CED-9 inhibits cell death by antagonizing the Apaf-1-like protein CED-4, which promotes death by activating CED-3 [8], [9]. CED-3 belongs to a cysteine protease family known as caspase [10]. It has been proposed that the binding of EGL-1 to CED-9 on the mitochondrial outer membrane transmits a pro-apoptotic signal that results in the CED-4-dependent activation of the cytoplasmic CED-3 caspase, thereby triggering apoptosis [8], [11]. Recent structural evidence suggests that eight CED-4 molecules form a funnel-shaped structure with four-fold symmetry, with each unit being defined by an asymmetric CED-4 dimer [12]. The cavity of this octameric structure provides space for two CED-3 molecules and facilitates their autocatalytic activation. Additionally, the auto-activation of the CED-3 zymogen is negatively regulated by the CED-3 orthologs CSP-2 and CSP-3, which lack caspase activity [13], [14], revealing that the regulation of CED-3 activity during programmed cell death is complex.

Additional factors that regulate the cell killing process during *C. elegans* development have been reported. MAC-1, an AAA family ATPase, can bind to CED-4 *in vitro* and prevent programmed cell death [15]. ICD-1 and TFG-1, which are similar to human βNAC and TRK-fused gene, respectively, suppress CED-4-dependent, but CED-3-independent, cell death [16], [17]. In contrast to these cell-death inhibitors, WAN-1, which is a mitochondrial adenine nucleotide translocator and is associated with CED-4 and CED-9 *in vitro*, can induce ectopic cell death dependently on the core cell death proteins [18]. It is not clear whether additional component(s) may exist to promote the cell killing process upstream of CED-3. Moreover, some cell death effectors that act downstream of (or in parallel to) CED-3, such as CED-8 [19] and WAH-1 [20], or are CED-3 substrates, such as DCR-1 [21], are important for the timing or progression of programmed cell death.

The eukaryotic translation initiation factor 3 (eIF3) plays essential roles in the initiation of translation [22]. The mammalian eIF3 complex contains 10–13 subunits, including five highly conserved core subunits and five to eight less conserved non-core subunits [23], [24]. The 28 kDa human eIF3k protein was originally identified as a non-core subunit of the eIF3 complex [25]. An *in vitro* reconstitution experiment showed that eIF3k is not required for the formation of the active eIF3 complex [26]. Interestingly, eIF3k is conserved among metazoans, including *C. elegans*, *D. melanogaster*, *M. musculus*, and *H. sapiens*, but is absent in *S. cerevisiae*, suggesting a specialized role for *eif-3.K* in multicellular organisms [25], [27]. In addition, human eIF3k is associated with dynein [27], cyclin D3 [28], the 5-HT_7_ receptor [29], and keratin K18 [30], suggesting the involvement of eIF3k in processes that are unrelated to translation. Recently, we reported an apoptosis-promoting function for eIF3k in simple epithelial cells [30]. Upon apoptotic stimuli, keratin K18 is cleaved by caspase 3, resulting in the collapse of K8/K18 intermediate filaments into apoptotic bodies and the sequestration of caspase 3 in kerain-containing inclusions [31]. eIF3k binds to keratin inclusions, which in turn leads to the release of keratin-associated caspase into the cytosol to facilitate the execution of apoptosis [30]. Keratin K8/K18 is the major intermediate filament in epithelial cells [31]. It is not clear whether eIF3k may potentiate apoptosis in other cell types, such as neurons or muscle cells, where intermediate filaments other than keratin are present. In addition, it is unclear whether the apoptosis-promoting function of eIF3k has been conserved throughout evolution.

In this work, we characterized the function of *eif-3.K* in *C. elegans* and showed that its apoptosis-promoting function has indeed been conserved throughout evolution. Furthermore, we identified a new function for the WH domain of EIF-3.K in the promotion of programmed cell death.

## Materials and Methods

### Strains

All strains were maintained at 20°C on NGM (nematode growth medium) agar seeded with *Escherichia coli* OP50 bacteria as previously described [32]. The wild-type strain was the Bristol strain N2. The following mutations were used: linkage group (LG) I, *ced-1(e1735)* [33], *csp-3(tm2486)* [14]; LGII, *icd-1(tm2873)* [17] *mIn1[dpy-10(e128)mIs14]*; LGIII, *ced-7(n1996)* [33], *ced-4(n1162*, *n2273)* [4], [34], *ced-6(n2095)* [33]; LGIV, *ced-5(n1812)*, *ced-2(n1994)* [33], *ced-3(n717*, *n2427)* [4], [35], *csp-2(tm3077)* [13]; LGV, *eif-3.K(gk126)* (*C. elegans* knockout consortium); *unc-76(e911)* [36]; *nuc-1(e1392)* [37]; LGX, *nIs106* [38]. The following integrated lines were used: *nIs50[P_mec-7_ced-3*A*]* [35], *bzIs8[P_mec-4_GFP]* [39] and *smIs1[P_mec-7_acCED-3; P_mec-3_GFP]* [40].

### Cell Death Assays

Cell corpse numbers in embryos or germline of indicated mutants were scored as previously described [41]. Extra surviving cells in the anterior pharynx were scored at the late L3 or early L4 larval stage, as previously described [41]. To assay extra surviving cells in the ventral cord, the integrated *nIs106* (*P_lin-11_gfp)* transgene was utilized [38]. The *nIs106* transgene was crossed to *ced-2* or *ced-7* single mutants or *ced-2; eif-3.K* or *ced-7; eif-3.K* double mutants. The extra Pn.aap cells in the P2, P9–P12-derived regions of the transgenic mutants were scored at the L4 stage by the fluorescence microscopy as previously described [38]. The TUNEL assay was carried out using an *in situ* cell-death detection kit (Roche) as previously described [42]. To assay the UV-C radiation-induced cell death in the germline, adult worms (24 h post the L4 stage) were exposed to 254 nm UV-C light at150 J/m^2^ using a Stratalinker UV crosslinker (Stratagene, model 2400) as previously described [43], and the cell corpses in the gonadal arms were scored 24 hours after the treatment.

### Molecular Biology

To determine the 5′ end of *eif-3.K* mRNA, we performed an RT-PCR experiment using nested primers 5′-GATGAGACACTTGGCGAGAG-3′ and 5′-CTTGTTTTCATTGACCATAGC-3′ in combination with either the SL1 primer or SL2 primer and sequenced the resulting product. The sequence confirmed the 5′ end of the *eif-3.K* coding sequence shown on the Wormbase and revealed that *eif-3.K* mRNA was trans-spliced to either SL1 or SL2. To generate the *eif-3.K* cDNA construct, the full-length *eif-3.K* coding region was amplified by RT-PCR using primers 5′-ATGTCGTTCGAGAAACTG-3′ and 5′-GTAAGTTGGGGGCAACTGAGAAATT-3′ and subsequently inserted into the pSTBlue vector (Novagen) at the *Eco*RV site. To generate *P_hsp_eif-3.K*, the *eif-3.K* cDNA was inserted into heat shock vectors pPD49.78 and pPD49.83 (different tissue specificity). To generate *P_let-858_eif-3.K* or *P_mec-4_eif-3.K*, *eif-3.K* was inserted to the pPD118.25 plasmid containing *P_let-858_* [44] or the pPD95.77 plasmid containing *P_mec-4_* [45], respectively. We generated mutant *eif-3.K* cDNA encoding truncated EIF-3.K protein without the HAM domain (amino acids 23–120) or WH domain (amino acids 148–208) by inverse PCR and inserted the mutant cDNA into the vector containing *P_let-858_* to generate *P_let-858_eif-3.K*ΔHAM or *P_let-858_eif-3.K*ΔWH, respectively. *eif-3.K*ΔWH cDNA was also inserted into vectors pPD49.78 and pPD49.83 to yield *P_hsp_eif-3.K*ΔWH. To generate *P_let-858_*WH or *P_hsp_*WH, the cDNA corresponding to the WH domain (amino acids 148–208) was inserted into the vector containing *P_let-858_* or *P_hsp_*, respectively. The *egl-1* [5] or *ced-4* cDNA [34] was cloned into pPD52.102 (Andy Fire) to generate *P_mec-7_egl-1* or *P_mec-7_ced-4*, respectively.

### Transgenic Animals

Germline transformation experiments were performed as previously described [46]. For the rescue experiment or structure function analysis of EIF-3.K, the indicated constructs (50 µg/ml) were injected into *eif-3.K(gk126)* animals with the coinjection marker pTG96 plasmid. The pTG96 plasmid contains *sur-5::GFP* that is expressed in almost all somatic cells [47].

To overexpress *egl-1*, *ced-4*, or *eif-3.K* in the touch neurons, *P_mec-7_egl-1*, *P_mec-7_ced-4*, or *P_mec-7_eif-3.K* (50 µg/ml) was injected into *unc-76(e911); bzIs8* animals with the coinjection marker p76–16B (100 µg/ml), which rescues the *unc-76* phenotype [36].To overexpress *ced-3* in touch neurons in the *bzIs8* transgenic worms, *nIs50* carrying the integrated transgene *P_mec-7_ced-3* (*ced-3*A line) [35] was crossed to the *bzIs8* strain to generate *nIs50; bzIs8* double transgenic worms. To express acCED-3 in touch neurons, the integrated transgene *smIs1* [40] carrying both *P_mec-7_acCED-3* and *P_mec-3_gfp* was used [40]. To coexpress *eif-3.K* and *ced-3* in touch neurons *P_mec-7_eif-3.K* (50 µg/ml) was injected into *bzIs8; nIs50* animals with the coinjection marker *P_myo-2_gfp* (2 µg/ml*)*, which expresses GFP in the pharynx [48].

### Heat Shock Experiments

To overexpress the wild-type or mutant *eif-3.K* cDNA or human eIF3k cDNA, young adults carrying the respective transgene were allowed to lay eggs overnight, and the laid embryos were cultured at 20°C (non-heat shock) or at 33°C (heat shock) for 1 hr, which was followed by a 20°C recovery for at least 1.5 hrs. The embryos were scored for cell corpses at the comma and 1.5-fold stages under DIC optics.

### Antibodies, Immunostaining and Immunoblotting

To generate anti-EIF-3.K antibodies, the *eif-3.K* cDNA corresponding to 45–240 amino acids was cloned into the pGEX-4T expression vector. GST- EIF-3.K(45–240) fusion protein was expressed in *E. coli* and further purified using 10% SDS-PAGE. GST-EIF-3.K(45–240) protein was excised from the gel and used to immunize rabbits. Immune serum was further purified by EIF-3.K-conjugated Affi-Gel as described by the manufacturer’s manual (Bio-Rad).

For immunostaining, embryos and worms were collected off plates and treated with hypochlorite (10 N NaOH and NaOCl) to enrich embryos. Embryos were then washed with ddH_2_O for three times and fixed in fixation buffer (2% paraformaldehyde, 90% methanol, 10% EGTA, 1 M spermine, 100 mM spermidine, and 0.5 M PIPES) overnight at -80°C as described by Guenther and Garriga [49]. After fixation, embryos were thawed, washed with Tris-Triton buffer (100 mM Tris-HCl pH7.4, 1% Triton X-100, and 1 mM EDTA) and blocked with 5% bovine serum albumin in PBS. Treated embryos were incubated with purified antibodies against EIF-3.K overnight at 4°C. After washing with wash buffer (1X PBS, 1% BSA, 0.5% Triton X-100, 0.05% NaN_3_, and 1 mM EDTA), embryos were then incubated with rhodamine-conjugated donkey secondary antibodies against rabbit (Jackson Immune Research Laboratories). After incubation for 2 hr at room temperature, antibodies were washed off using wash buffer three times for 5 min each, with DAPI included in the first wash. For MitoTracker® staining, embryos were collected from worms grown in the dark on NGM agar plates containing MitoTracker® Red 580 (1 µg/mL, Molecular Probes). Stained embryos were mounted with VECTASHIELD® mounting medium H-1000 (Vector Laboratories) and observed using confocal laser scanning microscopy (Leica TCS SP2 Confocal Spectral Microscope).

For western blot analysis, total protein extracts of indicated genotypes were resolved by 12% SDS-PAGE and transferred to nitrocellulose membranes. The blot was incubated with affinity-purified EIF-3.K antibodies (1∶2500) and monoclonal anti-α tubulin antibodies (Abcam). ECL detection system (pierce) was used for detection.

### Bacteria-mediated RNAi

Induction RNA interference (RNAi) experiments were carried out using a bacterial feeding protocol [50]. L4 larvae were transferred to the control (pPD129.36) or indicated RNAi plates and cultured at 20°C. F1 embryos laid approximately 48 hours later were picked for phenotypic analysis. The *eif-3.K* RNAi clone was obtained from the Ahringer RNAi library.

## Results

### The Loss of *eif-3.K* Causes Reduced Cell Death in Both Somatic and Germline Cells

**Figure 1:**
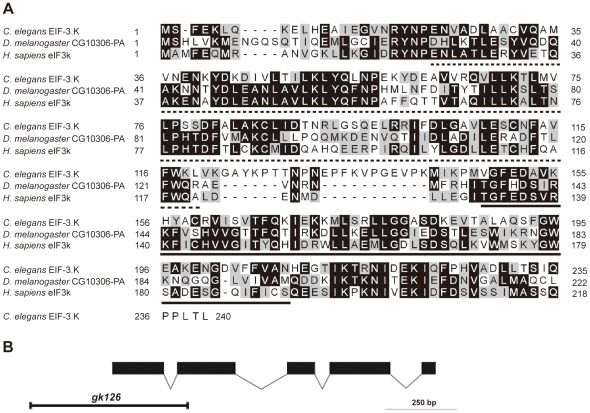
*eif-3.K* has been conserved throughout evolution. (A) An alignment between the EIF-3.K protein sequence and the *H. sapiens* eIF3k and *D. melanogaster* CG10306-PA protein sequences. Amino acids shaded in black are identical, and amino acids shaded in gray are similar. The predicted HAM and WH domains are underlined with dotted and black lines. (B) The gene structure of *eif-3.K* was deduced by comparing the coding sequence and the genomic DNA sequence. Boxes represent exons and lines between boxes represent introns. Solid boxes indicate the *eif-3.K* open reading frame. Open boxes indicate the untranslated region. The transcription direction is from left to right. The deleted region present in the *gk126* allele is indicated.

**Figure 2:**
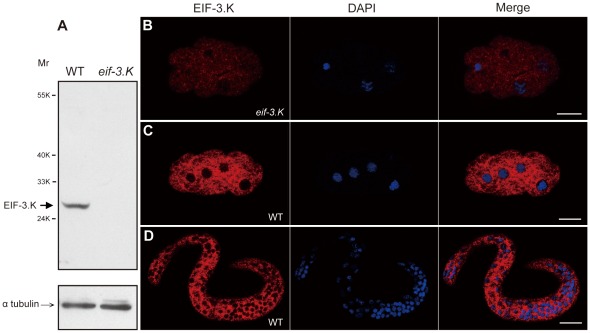
EIF-3.K protein expression. (A) Western blot analysis of EIF-3.K protein expression. Affinity-purified anti-EIF-3.K antibodies were used to probe a blot of embryonic extracts from wild-type and *eif-3.K(gk126)* worms (above). Equal loading of the two extracts was confirmed by anti-α tubulin antibodies (below). The sizes of molecular weight markers and the positions of EIF-3.K and α tubulin are indicated. (B–D) Images of an *eif-3.K(gk126)* mutant early embryo (B), a wild-type early embryo (C) and a wild-type newly hatched larva (D) that were co-stained with anti-EIF-3.K antibodies and DAPI. Merged images are also shown. Scale bar = 10 µm.

We obtained the cDNA for *eif-3.K* through reverse transcription polymerase chain reaction (RT-PCR) using total RNA from mixed-stage worms. The predicted full length amino acid sequence of EIF-3.K is 35% identical and 57% similar to that of human eIF3k (Figure 1A). We next characterized the *eif-3.K* mutant allele *gk126*, which was isolated by the *C. elegans* Gene Knockout Consortium. This allele contains a 538 base pair (bp) long deletion from 119 bp upstream of the start ATG codon to the second exon in the *eif-3.K* locus (Figure 1B). No EIF-3.K protein was detected in the *eif-3.K(gk126)* mutant by western blotting or immunostaining analyses using purified anti-EIF-3.K antibodies (Figure 2), suggesting that the *gk126* allele is null. Because *eif-3.K(gk126)* and *eif-3.K(RNAi)* mutant worms were viable and had normal development and growth rates (Table S1), we concluded that *eif-3.K* is not an essential component of the general translation machinery in *C. elegans*.

**Figure 3:**
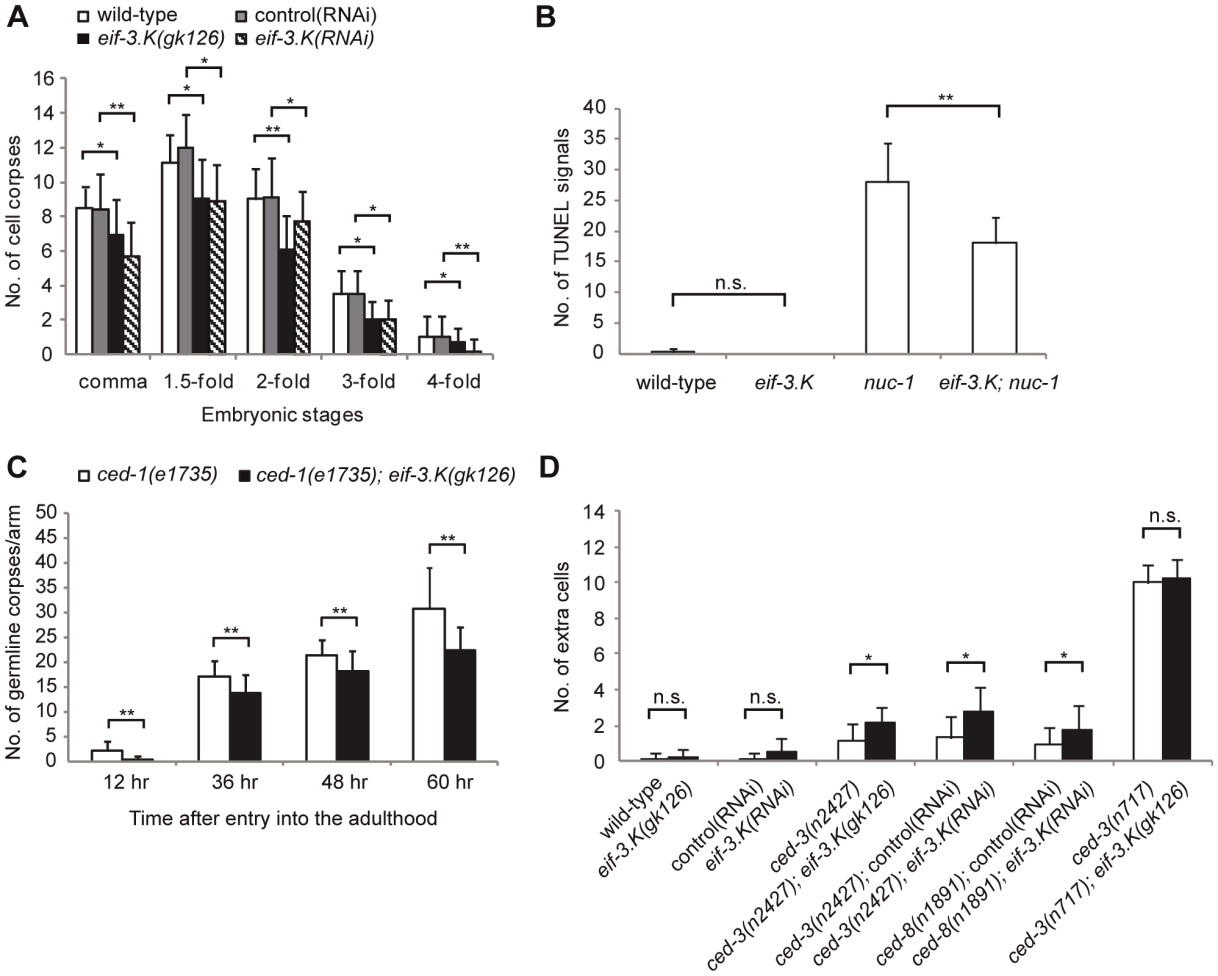
The loss of *eif-3.K* results in reduced programmed cell deaths. (A) The inactivation of *eif-3.K* by RNAi or by genetic deletion reduced cell corpse numbers throughout embryogenesis. Cell corpses of the indicated genotype or RNAi treatment were scored at the comma, 1.5-fold, 2-fold, 3-fold and 4-fold embryonic stages. The *eif-3.K(gk126)* embryos were compared to the wild-type embryos, and the *eif-3.K(RNAi)* embryos were compared to control(RNAi) embryos at each stage. All comparisons were performed using the unpaired t test (*P<0.05, **P<0.001). Data are presented as the mean ± standard deviation. Error bars represent S.D. Greater than 20 embryos per stage were analyzed. (B) The loss of *eif-3.K* reduces TUNEL staining in *nuc-1* embryos. The number of cells exhibiting TUNEL staining was determined in embryos of the indicated genotypes at the 1.5-fold stage. The *eif-3.K(gk126)* embryos were compared to the wild-type embryos, and the *nuc-1(e1392)*single mutants were compared to the *eif-3.K(gk126); nuc-1(e1392)* double mutants. Comparisons were performed using the unpaired t test (*P<0.05, **P<0.001). Data are presented as the mean ± standard deviation. Error bars represent S.D. Greater than 20 embryos of each genotype were analyzed. n.s. indicates no significant difference. (C) A loss of *eif-3.K* reduces cell corpse number in the germline. Cell corpses in the germline of the *ced-1* single mutants (white columns) and *eif-3.K; ced-1* double mutants (black columns) were counted at the indicated times after entry into the adulthood. The y axis represents the average number of cell corpses scored in each gonadal arm. The *eif-3.K(gk126); ced-1(e1735)* double mutants were compared to the *ced-1(e1735)* single mutants at the same developmental stage using the unpaired t test (*P<0.05, **P<0.001). Data are presented as the mean ± standard deviation for >20 gonadal arms. Error bars represent S.D. (D) A loss of *eif-3.K* increases the number of extra surviving cells in weak *ced-3* mutants. Cells that failed to undergo programmed cell deaths in the anterior pharynx were scored in the indicated animals. All comparisons were performed using the unpaired t test (*P<0.05, **P<0.001). Data are presented as the mean ± standard deviation for >20 larvae. Error bars represent S.D. n.s. indicates no significant difference.

We next examined whether *eif-3.K(RNAi)* or *eif-3.K(gk126)* embryos have defective programmed cell death. A time course analysis of embryonic cell corpses using differential interference contrast (DIC) microscopy showed that *eif-3.K(RNAi)* or *eif-3.K(gk126)* embryos had fewer cell corpses than wild-type embryos throughout embryogenesis (Figure 3A). To determine whether this decrease in cell corpse number corresponded with a reduction in cell death or was simply due to abnormal corpse morphology, we further analyzed the embryos using the TUNEL (Terminal deoxynucleotidyl transferase dUTP nick end labeling) assay. The degradation of DNA in dying cells is a hallmark of apoptosis and can be detected *in situ* using TUNEL staining [42], [51]. As shown previously [42], wild-type embryos had very few TUNEL-positive corpses (Figure 3B); however, embryos lacking the *nuc-1* gene, which codes for a protein similar to DNAse II that is involved in DNA degradation [42], had many more TUNEL-positive corpses (Figure 3B). The *eif-3.K* embryos, like the wild-type embryos, had few TUNEL-positive corpses; however, *eif-3.K; nuc-1* double mutant embryos had fewer TUNEL-positive corpses than *nuc-1* single mutant embryos, indicating that apoptotic.

DNA degradation is compromised in the *eif-3.K* mutants. This result, together with the observed decrease in cell corpse number (Figure 3A), indicates that apoptosis is compromised in *eif-3.K* mutants during embryogenesis.

Like somatic cells, germline cells also undergo apoptosis in *C. elegans* [52]. Because few germ cell corpses can be observed in the wild-type adult gonad at any given time due to the prompt removal of cell corpses by the gonadal sheath cells [52], we utilized *ced-1(e1735)* mutant worms, in which cell corpses are not efficiently removed and therefore accumulate, to increase our chances of detecting cell corpses. We found that *ced-1(e1735); eif-3.K(gk126)* double mutants had significantly fewer germ cell corpses than *ced-1(e1735)* single mutants at all adult stages (Figure 3C). Therefore, *eif-3.K* is also important for programmed cell death in germline cells.

### The Loss of *eif-3.K* Enhances Cell Survival in Sensitized Mutants

We next examined whether a loss of *eif-3.K* function could prevent cell death and result in an accumulation of surviving cells. Two assays were used to score the surviving cells in various regions of the animal [53]. First, superfluous surviving cells that were present in the anterior pharynx were scored using DIC optics. As was previously shown [54], in the presence of a strong loss-of-function mutation in the pro-apoptotic gene *ced-3(n717)*, which blocks nearly all cell deaths, resulted in approximately 10 additional surviving cells in the anterior pharynx (Figure 3D). Animals harboring the weak *ced-3(n2427)* mutation had only 1.2 additional surviving cells (Figure 3D). We found that the *eif-3.K(RNAi)* or *eif-3.K(gk126)* single mutant animals had 0.2 or 0.5 extra surviving cells, similar to the wild-type animals (Figure 3D), indicating that the loss of *eif-3.K* could not detectably block apoptosis in these cells; however, the *eif-3.K(RNAi)* or *eif-3.K(gk126)* mutation did enhance cell survival in the weak *ced-3(n2427)* mutant animals. The *ced-3(n2427); eif-3.K(RNAi)* or *ced-3(n2427); eif-3.K(gk126)* double mutants had 2.7 or 2.1 additional surviving cells in the anterior pharynx (Figure 3D). This is significantly more than *ced-3(n2427)*, *eif-3.K(RNAi)* or *eif-3.K(gk126)* single mutants. Moreover, the RNAi-mediated inactivation of *eif-3.K* also significantly enhanced cell survival in the worms lacking the *ced-8* gene (Figure 3D), which controls the timing of programmed cell death [19]. These results show that the loss of *eif-3.K* enhances cell survival in sensitized mutants.

We further analyzed the identities of the surviving cells in these mutants. The extraneous surviving cells observed in *ced-3(n2427)* single mutants and *ced-3(n2427); eif-3.K(gk126)* double mutants appeared similar and included sisters of muscle cells m1and m2 and neurons I1, I2, and MC (Figure S1). It is possible that these cells are more likely to survive than others when the apoptotic machinery is compromised. Consistent with this hypothesis, m1 and m2 sister cells were occasionally observed to survive in the wild-type or *eif-3.K(gk126)* animals (Figure S1).We also compared the identities of surviving cells in the *ced-3(n2427)* animals that were treated with either the *eif-3.K* or control RNAi. Compared to the control RNAi, *eif-3.K* RNAi enhanced the survival of the niece of the epithelial cell e1, the sister of the neuron I1 and those cells that were also enhanced by the *eif-3.K(gk126)* mutation in the *ced-3(n2427)* single mutants, including sisters of m1, m2, I1, I2, and MC cells (Figure S1). Because the *eif-3.K* null allele did not enhance the total number of extra surviving cells in the strong *ced-3(n717)* mutants (Figure 3D), *eif-3.K* likely functions with *ced-3* in the same genetic pathway to promote most, if not all, programmed cell death. Additionally, because the identities of apoptotic cells can be inferred from the cell fates of their differentiated sister cells [55], our observations suggest that *eif-3.K* exerts a cell death-promoting function in multiple cell types, including neuron, muscle and epithelial cells during development.

Secondly, we scored superfluous surviving cells in the ventral cord in larvae. In contrast to the extra surviving cells we observed in the anterior pharynx, which are generated during embryogenesis [56], extra surviving cells in the ventral cord are generated during larval development [57]. In strong *ced-3(n717)* mutants, five cells P2.aap and P9–P12.aap in the ventral cord survive [38]. These Pn.aap cells are known to differentiate into VC motor neuron-like cells and express the *P_lin-11_gfp* reporter (Table 1) [38]. We scored extra surviving Pn.aap cells using the *P_lin-11_gfp* transgene as a marker and found that only 2% of *eif-3.K(gk126)* worms exhibited extra Pn.aap cells (Table 1). However, the *eif-3.K(gk126)* mutation increased the average number of extra surviving Pn.aap cells in *ced-3(n2427)* mutants from 2.6 to 3.6 (Table 1). A previous study showed that strong mutations in genes essential for the removal of apoptotic cells, such as *ced-2* or *ced-7*, block cell death, albeit at low efficiency, as apoptotic cell removal is involved in the death of cells [38], [58]. The frequency of extra Pn.aap cell survival in these mutants can be enhanced by a weak mutation in the core programmed cell death genes *ced-3*, *ced-4*, or *egl-1* [38]. Therefore, we tested whether the loss of *eif-3.K* enhanced the frequency of superfluous Pn.aap cell survival in strong *ced-2* or *ced-7* mutants. We found that the *eif-3.K(gk126)* mutation increased the frequency of Pn.aap cell survival in *ced-2 (n1994)* or *ced-7(n1996)* mutants from 27% to 100% and 83% to 93%, respectively (Table 1). In addition, the average number of extra surviving Pn.aap cells in *ced-2(n1994)* or *ced-7(n1996)* mutants also increased from 0.3 to 2.4 and 1.6 to 2.1, respectively (Table 1). These observations support the idea that the cell death machinery is compromised in the *eif-3.K* mutants. The observed decreased cell death in *eif-3.K* mutants (Figures 3A–3C) as well as the enhanced cell survival observed in the anterior pharynx (Figure 3D) and ventral cord (Table 1) of sensitized mutants shows that *eif-3.K* is a positive mediator of programmed cell death.

**Table 1:** The loss of *eif-3.K* enhances cell survival in the ventral cord of sensitized mutants.

Transgene	Genotype	Average number of extra Pn.aap cells^a^	Animals with surviving Pn.aap cells^b^ (%)
*P_lin-11_gfp*	wild-type	0.0	0
*P_lin-11_gfp*	*ced-3(n717)*	5.0	100
*P_lin-11_gfp*	*ced-3(n2427)*	2.6	99
*P_lin-11_gfp*	*eif-3.K(gk126)*	0.0	2
*P_lin-11_gfp*	*ced-3(n2427); eif-3.K(gk126)*	3.6	100
*P_lin-11_gfp*	*ced-2(n1994)*	0.3	27
*P_lin-11_gfp*	*ced-2(n1994); eif-3.K(gk126)*	2.4	100
*P_lin-11_gfp*	*ced-7(n1996)*	1.6	83
*P_lin-11_gfp*	*ced-7(n1996); eif-3.K(gk126)*	2.1	93

^a^Average numbers of fluorescent cells caused by expression from *P_lin-11_gfp* in P2, 9, 10, 11, and 12-derived regions were determined using DIC microscopy equipped with an ultraviolet light source. Greater than 20 larvae of each genotype were analyzed.

b The percentages of animals that had at least one fluorescent cell in the P2, 9, 10, 11, and 12-derived regions were determined.

### The Loss of *eif-3.K* Partially Suppresses the Ectopic Cell Deaths Induced by the Overexpression of *egl-1* or *ced-4*

**Figure 4:**
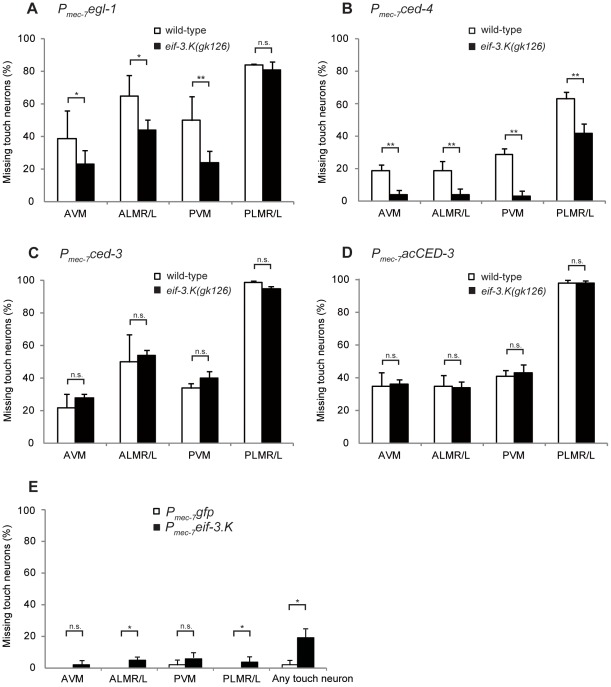
The loss of *eif-3.K* partially suppresses cell death induced by the overexpression of *egl-1*and *ced-4,* but not *ced-3*. (A–D) The percentage of animals missing specific touch neurons are shown for the wild-type (white columns) or *eif-3.K(gk126)* (black columns) embryos carrying the *P_mec-7_egl-1* (A), *P_mec-7_ced-4* (B), *P_mec-7_ced-3* (C), or *P_mec-7_acCED-3* (D) transgenes. The *eif-3.K(gk126)* transgenic worms were compared to the analogous wild-type transgenic worms. Comparisons were performed using the unpaired t test (*P<0.05, **P<0.001). Data are presented as the mean ± standard deviation. Error bars represent S.D. n.s. indicates no significant difference. More than 100 animals were scored for each strain. (A)The percentage of animals missing specific touch neurons or missing at least one touch neuron are shown for wild-type control *P_mec-7_gfp* transgenic animals (white columns) or *P_mec-7_eif-3.K* transgenic animals (black columns). More than 100 animals were scored for each strain.

We next tested whether *eif-3.K* genetically interacts with the core programmed cell death genes *ced-3*, *ced-4*, and *egl-1.* Previous studies have shown that cell-specific expression of these three genes under the control of the *P_mec-7_* promoter, which is expressed in six touch neurons (AVM, ALMR/L, PVM, and PLMR/L), promotes these neurons to undergo programmed cell death [5], [35]. We tested if the programmed cell death of these cells required the activity of *eif-3.K*. To facilitate the scoring of touch neurons, the *P_mec-4_gfp* reporter in the integrated transgene *bzIs8*, which labels the six touch neurons with GFP, was used as a cell viability marker [39]. We found that the *eif-3.K(gk126*) mutation partially suppressed the apoptosis of the touch neurons that was induced by the overexpression of *egl-1* or *ced-4* (Figures 4A and 4B). For example, the overexpression of *egl-1* or *ced-4* resulted in the death of approximately 50% or 29% of the PVM neurons, respectively. The loss of *eif-3.K* reduced cell death to 24% and 3%, respectively. A significant reduction in cell death was also observed in other touch neurons, except for PLMR/L with *egl-1* overexpression (Figure 4A and 4B). Therefore, the efficient apoptosis of touch neurons induced by the overexpression of *egl-1* or *ced-4* requires *eif-3.K*. This result showed that *eif-3.K* functions downstream of or in parallel to *egl-1* or *ced-4* to promote cell death.

In contrast, *eif-3.K(gk126*) failed to suppress the apoptosis of touch neurons induced by *ced-3* overexpression. The overexpression of *ced-3* resulted in the death of approximately 34% of the PVM neurons in the wild-type animals, similar to the percentage (40%) of cell death observed in the *eif-3.K(gk126)* mutants (Figure 4C). When activated CED-3 (acCED-3) was expressed in touch neurons via the *P_mec-7_acCED-3* transgene [40], 40% and 43% of the PVM neurons were killed in the wild-type and *eif-3.K(gk126)* worms, respectively (Figure 4D). This result showed that *eif-3.K* also fails to inhibit apoptosis caused by the overexpression of activated CED-3. Similar results were observed in other touch neurons expressing either the *P_mec-7_ced-3* or the *P_mec-7_acCED-3* transgene (Figures 4C and 4D).

We next examined if overexpression of *ced-3* using the heat shock promoter *P_hsp_* was able to rescue the cell death defects caused by the *eif-3.K(gk126)* mutation. Heat shock-induced *ced-3* overexpression rescued the defect at the comma and 1.5-fold stages, but it also slightly elicited ectopic cell killing at the comma stage (Table 2). The *eif-3.K(gk126)* embryos carrying the *P_hsp_ced-3* transgene had approximately 7.0 cell corpses at the comma stage under non-heat shock condition. The heat shock-induced overexpression of *ced-3* in the transgenic embryos at the same developmental stage increased the cell corpse number to 10.7, which was significantly more than the 8.4 cell corpses that were observed in the wild-type embryos carrying the control *P_hsp_gfp* transgene under the same conditions (Table 2). This result supports the model that *ced-3* acts downstream of *eif-3.K* to execute programmed cell death.

**Table 2:** Overexpression of *eif-3.K* or *ced-3* in cell death-defective mutants.

			No. of Cell Corpses^b^	
Transgene	Genotype	Heat shock^a^	comma	1.5-fold
*P_hsp_gfp*	wild-type	–	8.0±0.9	10.8±1.2
*P_hsp_gfp*	wild-type	+	8.4±1.1	11.1±1.5
*P_hsp_gfp*	*ced-3(n717)*	–	0.0±0.0	0.0±0.0
*P_hsp_gfp*	*ced-3(n717)*	+	0.0±0.0	0.0±0.2
*P_hsp_gfp*	*ced-4(n1162)*	–	0.0±0.0	0.0±0.2
*P_hsp_gfp*	*ced-4(n1162)*	+	0.1±0.3	0.1±0.1
*P_hsp_gfp*	*eif-3.K(gk126)*	–	6.7±2.2	9.0±2.1
*P_hsp_gfp*	*eif-3.K(gk126)*	+	6.8±2.0	8.9±1.9
*P_hsp_ced-3*	wild-type	–	7.8±1.1	10.8±1.6
*P_hsp_ced-3* c	wild-type	+	11.2±2.1**	12.8±2.2**
*P_hsp_ced-3*	*eif-3.K(gk126)*	–	7.0±1.8	8.9±2.0
*P_hsp_ced-3* c	*eif-3.K(gk126)*	+	10.7±0.6**	11.6±1.7**
*P_hsp_eif-3.K*	wild-type	–	8.5±2.1	11.2±2.4
*P_hsp_eif-3.K*	wild-type	+	9.4±1.7	13.4±2.9*
*P_hsp_eif-3.K*	*ced-3(n717)*	–	0.0±0.0	0.0±0.2
*P_hsp_eif-3.K*	*ced-3(n717)*	+	0.2±0.4	0.4±0.5*
*P_hsp_eif-3.K*	*ced-4(n1162)*	–	0.1±0.3	0.0±0.2
*P_hsp_eif-3.K*	*ced-4(n1162)*	+	0.2±0.4	0.1±0.2

a Transgenic animals were subjected to heat-shock (+) or left at 20°C (−).

b Transgenic embryos were scored for the number of cell corpses 1.5 hrs after heat shock (see Materials and methods). Data are presented as the mean ± standard deviation from two independent stably transmitting lines.

c Greater than 20 embryos were analyzed from each line except for 1.5-fold embryos carrying *P_hsp_ced-3* (n≥8) after heat shock due to high lethality.

The transgenic embryos after heat shock were compared to the corresponding transgenic embryos without heat shock. All comparisons were performed using the unpaired t test (*P<0.05, **P<0.001).

### *eif-3.K* Acts Upstream of *ced-3* in the Promotion of Programmed Cell Death

To determine whether *eif-3.K* promotes programmed cell death, we tested if the overexpression of *eif-3.K* caused cells that would normally live to undergo programmed cell death by overexpressing *eif-3.K* under the control of the heat shock promoter *P_hsp_* in the wild-type animals. The overexpression of *eif-3.K*, but not of the control *gfp*, slightly but significantly increased the cell corpse number at the 1.5-fold stage, despite a lack of significant ectopic killing at the comma stage (Table 2). Nonetheless, this ectopic killing at the 1.5-fold stage supports for a cell death-promoting function for *eif-3.K*. In addition, this ectopic killing was significantly suppressed by the strong *ced-3(n717)* or *ced-4(n1162)* mutations (Table 2). This result, in combination with the reciprocal experiment in which the loss of *eif-3.K* suppressed the efficient apoptosis of touch neurons in the presence of *ced-4* overexpression (Figure 4B), suggests a mutual requirement for *eif-3.K* and *ced-4.* Additionally, because the loss of *eif-3.K* failed to suppress the efficient apoptosis of touch neurons in the presence of *ced-3* overexpression, our result suggests a unidirectional requirement of *eif-3.K* for *ced-3* to achieve effective ectopic cell death under overexpression conditions.

### *eif-3.K* Promotes Cell Death in a Cell-Autonomous Fashion

To determine whether *eif-3.K* promotes programmed cell death in a cell-autonomous fashion, we tested if the *P_mec-7_eif-3.K* transgene, in which *eif-3.K* is expressed under the *P_mec-7_* promotor in touch neurons, could trigger touch neuron apoptosis in the wild-type animals. Although the overexpression of *eif-3.K* transgene resulted in a low frequency of individual touch neuron apoptosis, approximately 19.2% of trasngenic worms had at least one missing touch neuron (Figure 4E). In contrast, only 1.9% of the wild-type animals carrying the control *P_mec-7_gfp* transgene had one missing touch neuron (Figure 4E). This result not only reinforced the cell death-promoting function for *eif-3.K* but also showed that *eif-3.K* executes this function in a cell-autonomous fashion.

### The Loss of *eif-3.K* Significantly Reduces Ectopic Cell Deaths in *icd-1* Mutants

**Figure 5:**
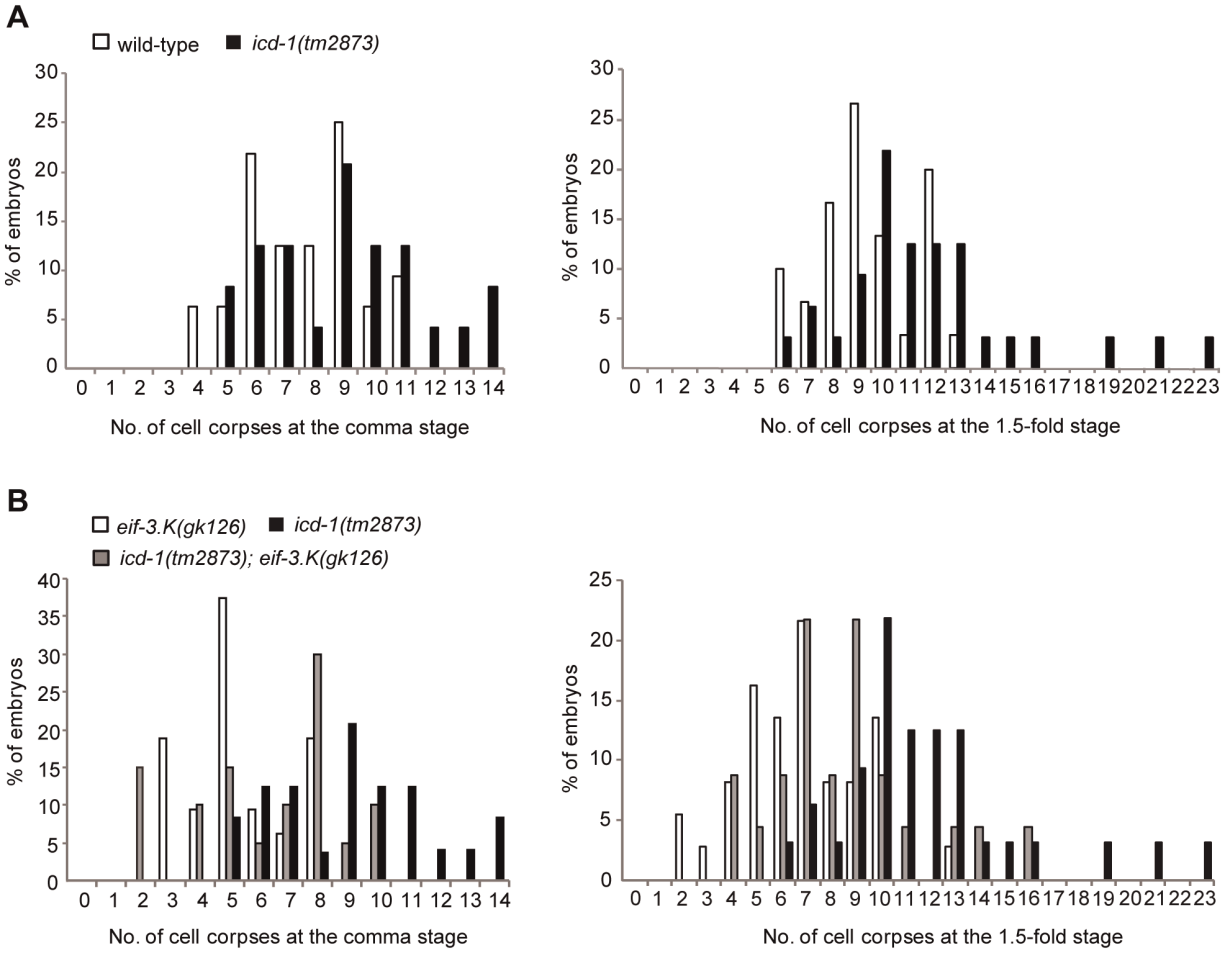
*eif-3.K* partially suppresses the ectopic cell deaths caused by the loss of *icd-1*. (A) A quantification of the cell corpses present in the wild-type and *icd-1(tm2873)* embryos at the comma (left) and 1.5-fold (right) stages. (B) A quantification of the cell corpses present in the *eif-3.K(gk126)* and *icd-1(tm2873)* single mutant and *icd-1(tm2873); eif-3.K(gk126)* double mutant embryos at the comma (left) and 1.5-fold (right) stages. The y axis shows the percentage of embryos and the x axis shows the cell corpse number. More than 20 embryos for each genotype at each stage were scored.

The inactivation of *icd-1* (inhibitor of cell death-1) by RNAi results in ectopic cell death that can be blocked by the loss of *ced-4* but not *ced-3*, revealing that the cell death in *icd-1(RNAi)* embryos is *ced-4*-dependent but *ced-3*-indpendent [17]. The observation that *eif-3.K* is required for cell death induced by the overexpression of *ced-4* but not *ced-3* (Figures 4B–4D) prompted us to test whether *eif-3.K* could suppress ectopic cell death resulting from the loss of *icd-1*. Like the *icd-1(RNAi)* embryos described previously [17], *icd-1(tm2873)* embryos had additional cell corpses compared to the wild-type embryos at the comma and 1.5-fold stages (Figure 5A). Although the *eif-3.K(gk126)* mutation significantly reduced the cell corpse number in *icd-1(tm2873)* embryos at both stages, the cell corpse number was not reduced to the extent observed in the *eif-3.K(gk126)* mutants alone (Figure 5B). Instead, the cell corpse number for the double mutant was between that observed in the *eif-3.K(gk126)* and *icd-1(tm2873)* single mutants. This result is consistent with the model that *eif-3.K* acts in parallel with *icd-1* to promote cell death; however, we cannot rule out the possibility that *icd-1* may in part prevent programmed cell death in an *eif-3.K-*dependent manner.

### EIF-3.K is Widely Expressed throughout Embryogenesis and Localized to the Cytoplasm

To determine the localization pattern of EIF-3.K, we raised antibodies against a recombinant EIF-3.K protein (see Experimental Procedures). Using affinity-purified EIF-3.K antibodies and western blot analysis, we detected a band of apparent molecular mass 27 kDa from wild-type worm extracts by western blot analysis (Figure 2A). This protein was absent in extracts from the *eif-3.K(gk126*) mutants (Figure 2A), confirming that the 27 kDa protein is the product of the *eif-3.K* gene. We used the purified EIF-3.K antibodies to stain embryos and larvae. EIF-3.K was widely expressed in embryos and larvae and was localized to the cytoplasm (Figures 2B–2D). EIF-3.K did not appear to be associated with mitochondria, where several cell death regulators such as CED-9, WAH-1, and WAN-1 are located [7], [18], [20], because EIF-3.K did not co-localize with MitoTracker Red, a marker of mitochondria (Figure S2).

### The WH Domain of EIF-3.K is Necessary and Sufficient for its Cell Death-Promoting Activity

EIF3.K contains two distinct domains, the HAM (HEAT Analogous Repeats) and WH (Winged Helix) domains, which have been implicated in protein-protein and protein-RNA interactions, respectively [59]. To test the importance of these domains for EIF-3.K function, we deleted the region corresponding to the HAM or WH domains, respectively (*eif-3.K*ΔHAM or *eif-3.K*ΔWH constructs). We then tested the ability of the mutant construct to rescue the cell death defects in *eif-3.K(gk126)* embryos by expressing the mutant construct under the control of the ubiquitous *let-858* promoter *P_let-858_* [44]. To our surprise, the HAM domain, comprising more than one-third of the EIF-3.K protein, was dispensable for *eif-3.K* activity, as *P_let-858_eif-3.K*ΔHAM completely rescued the cell death defect in the *eif-3.K(gk126)* embryos (Table 3). In contrast, *P_let-858_eif-3.K*ΔWH failed to rescue the defect (Table 3), suggesting an essential role for the WH domain in the cell death-promoting function of *eif-3.K*. Because the expression level of the *P_let-858_eif-3.K* transgene was lower than that of the endogenous *eif-3.K*, as detected by western blotting or immunostaining analysis (data not shown), the stronger heat shock promoter *P_hsp_* was subsequently used to increase the expression of the mutant *eif-3.K* construct in an effort to confirm our results. The heat shock-induced expression of the wild-type and mutant *eif-3.K* genes resulted in slightly higher protein expression levels (Figure S3). The overexpressed proteins exhibited a similar localization as the endogenous EIF-3.K protein, suggesting that these proteins localize normally (Figure S3). Similar to the results obtained using the *P_let-858_* promoter, expression under the heat shock promoters revealed that *eif-3.K*ΔHAM, but not *eif-3.K*ΔWH, rescued the *eif-3.K* mutant phenotype at the comma and 1.5-fold stages (Table 3). These data show that the WH domain, but not the HAM domain, is necessary for the cell death-promoting function of EIF-3.K.

**Table 3:** Structure and function analysis of *eif-3.K*.

			No. of Cell Corpses^b^	
Transgene	Genotype	Heat shock^a^	comma	1.5-fold
none	wild-type	–	8.4±0.9	11.3±1.0
none	*eif-3.K(gk126)*	–	6.9±2.1	9.0±2.3
*P_let-858_eif-3.K*ΔHAM	*eif-3.K(gk126)*	–	7.8±1.1*	11.0±1.0**
*P_let-858_eif-3.K*ΔWH	*eif-3.K(gk126)*	–	6.5±1.2	9.1±1.3
*P_let-858_*WH	*eif-3.K(gk126)*	–	8.1±0.7*	11.0±1.2**
*P_hsp_gfp*	*eif-3.K(gk126)*	–	6.7±2.2	9.0±2.1
*P_hsp_gfp*	*eif-3.K(gk126)*	+	6.8±2.0	8.9±1.9
*P_hsp_eif-3.K*	*eif-3.K(gk126)*	–	6.9±2.0	8.8±1.9
*P_hsp_eif-3.K*	*eif-3.K(gk126)*	+	8.8±1.2**	11.3±1.1**
*P_hsp_eif-3.K*	wild-type	–	8.5±2.1	11.2±2.4
*P_hsp_eif-3.K*	wild-type	+	9.4±1.7	13.4±2.9**
*P_hsp_eif-3.K*ΔWH	*eif-3.K(gk126)*	–	6.4±2.1	8.7±1.8
*P_hsp_eif-3.K*ΔWH	*eif-3.K(gk126)*	+	6.2±1.1	8.6±0.8
*P_hsp_eif-3.K*ΔHAM	*eif-3.K(gk126)*	–	6.5±2.0	8.8±2.0
*P_hsp_eif-3.K*ΔHAM	*eif-3.K(gk126)*	+	8.3±1.1**	11.2±1.9**
*P_hsp_*WH	*eif-3.K(gk126)*	–	6.5±2.1	9.0±1.8
*P_hsp_*WH	*eif-3.K(gk126)*	+	8.3±0.9**	10.9±1.0*
*P_hsp_*WH	wild-type	–	8.4±2.0	11.0±2.4
*P_hsp_*WH	wild-type	+	10.1±2.2*	13.1±2.1*
*P_hsp_*eIF3k(human)	*eif-3.K(gk126)*	–	6.5±1.9	8.6±1.7
*P_hsp_*eIF3k(human)	*eif-3.K(gk126)*	+	8.7±0.8**	10.4±0.6**

^a^Transgenic animals were subjected to heat-shock (+) or left at 20°C (−).

b Transgenic embryos were scored for the number of cell corpses 1.5 hrs after heat shock.

(see Materials and methods).

Data are presented as the mean ± standard deviation from two independent stably transmitting lines. Greater than 20 embryos were analyzed from each line.

For the *P_let-858_* expressing transgene, *eif-3.K* mutant embryos carrying the transgene were compared to *eif-3.K* mutant without the transgene.

For the *P_hsp_* expressing transgene, the transgenic embryos after heat shock were compared to the corresponding transgenic embryos without heat shock. All comparisons were performed using the unpaired t test (*P<0.05, **P<0.001).

We next tested if the WH domain of EIF-3.K is sufficient to rescue the cell-death defect caused by the *eif-3.K* mutation. We expressed the WH domain alone using either the *P_let-858_* or *P_hsp_* promoters in the transgenes *P_let-858_*WH or *P_hsp_*WH, respectively. We found that either promoter rescued the cell death defect in the *eif-3.K* mutants (Table 3). Moreover, when the *P_hsp_*WH transgene was expressed in the wild-type animals, it induced ectopic cell deaths. Furthermore, superfluous cell corpses were observed at the comma and 1.5-fold stages (Table 3). Therefore, the WH domain is both necessary and sufficient for the cell death-promoting activity of *eif-3.K*.

### Human eIF3k can Partially Substitute for *C. elegans* EIF-3.K

The human eIF3k mediates apoptosis in simple epithelial cells, likely by binding to keratin K18 via its HAM domain [30]; however, the HAM domain of *C. elegans* EIF-3.K appears dispensable for its function in cell death. We tested whether the expression of human eIF3k by *P_hsp_* was able to rescue the cell death defect caused by the *eif-3.K* mutation. We found that human eIF3k partially rescued the defective apoptosis in the *eif-3.K(gk126*) mutants (Table 3). This result indicates that the pro-apoptotic function of EIF-3.K has been conserved through evolution from *C. elegans* to humans and that the mechanisms by which human eIF3k and *C. elegans* EIF-3.K promote apoptosis may, in part, be similar.

## Discussion

eIF3 is the largest and most complex translation initiation factor, consisting of thirteen subunits in both *C. elegans* and humans [22]. The RNAi-based or genetic inactivation of ten eIF3 subunits, including *eif-3.A (egl-45)*, *eif-3.B*, *eif-3.C*, *eif-3.D*, *eif-3.E*, *eif-3.F*, *eif-3.G*, *eif-3.H*, *eif-3.J*, or *cif-1(eif-3.M)*, in *C. elegans* can cause sterility, embryonic lethality or gross developmental defects [60]–[63]. In contrast, the *eif-3.K* null mutant is viable and healthy (Table S1), suggesting that *eif-3.K* is not essential for general translation initiation. Similarly, human eIF3k is dispensable for the formation of an active eIF3 complex *in vitro* [26]. We have previously shown that human eIF3k promotes apoptosis in cultured simple epithelial cells [30]. In this work, we provide evidence that *eif-3.K* has a cell death-promoting function at an organismal level and that this function has been conserved through evolution.

In *C. elegans*, the loss of *eif-3.K* caused reduced programmed cell death (Figures 3A–3C) and enhanced cell survival in sensitized mutants (Figure 3D and Table 1). In contrast, the overexpression of *eif-3.K* by the heat shock promoter or a touch neuron-specific promoter resulted in ectopic cell death (Table 2 and Figure 4E). These results demonstrate that *eif-3.K* promotes programmed cell death. Our results also show that *eif-3.K* is essential for the efficient cell death that is induced by the overexpression of *egl-1* or *ced-4*, but not *ced-3*, as the loss of *eif-3.K* partially suppresses the cell death that is induced by the overexpression of *egl-1* or *ced-4* only (Figures 3A–3D). In addition, the observation that *ced-3* overexpression can rescue the cell death-defective phenotype of *eif-3.K* mutants and that the *ced-3* strong mutation can suppress cell death caused by heat shock-induced *eif-3.K* overexpression (Table 2) further reinforces the notion that *eif-3.K* requires *ced-3* to promote programmed cell death. Furthermore, the wide range in the identity and type of extraneous surviving cells that are affected by the *eif-3.K* mutation (Figure S1) suggests that *eif-3.K* may be involved in the majority of programmed cell death. This is consistent with the ubiquitous expression of EIF-3.K in embryos and larvae (Figures 2C and 2D). In addition to physiological cell deaths, DNA damage caused by genotoxic stress such as UV or IR radiation also induces cell deaths in the germline [43], [64]. We found that the *eif-3.K(gk126)* mutation significantly reduced UV-induced cell deaths in the germline (Figure S5), indicating that *eif-3.K* also mediates DNA damage-induced cell death.

During *C. elegans* development, the activity of the executioner caspase CED-3 is under both positive and negative regulation. Previous studies have shown that CED-4 facilitates the auto-cleavage of pro-CED-3 to generate the active CED-3 caspase during the promotion of cell death [12], [65], [66], while the CED-3 paralogs CSP-2 and CSP-3 associate with the CED-3 zymogen and inhibits its auto-activation, thereby protecting cells from inappropriate apoptosis [13], [14]. Our observation that neither EIF-3.K nor its WH domain bind to CED-3 or CED-4 in a yeast 2-hybrid system (Figure S4) suggests that EIF-3.K may not promote cell death through a direct association with either protein. In addition, since CED-3 and CED-4 are the only known proteins involved in CED-3 activation from pro-CED-3 [9], [12], EIF-3.K likely does not affect this activation process directly. It is possible that EIF-3.K may promote programmed cell death after CED-4-induced CED-3 activation. Human eIF3k has been proposed to promote apoptosis by facilitating the release of active caspases from an inhibitory compartment of intermediate filament-containing inclusions into the cytosol, thereby allowing the released caspase better access to its cytosolic substrates [30]. Although the mechanism by which eIF3k may affect the release of caspases from intermediate filament-containing inclusions is not clear, the binding of eIF3k to intermediate filaments is known to be important for the release process [30]. Similarly, *C. elegans* EIF-3.K may promote programmed cell death by affecting the distribution of active CED-3, thus facilitating the substrate cleavage and the subsequent execution of cell death. Alternatively, EIF-3.K might promote programmed cell death in parallel with CED-4 by antagonizing CSP-2 or CSP-3, thus facilitating CED-3 auto-activation from the zymogen in germline or somatic cells, respectively [13], [14]. To test the latter possibility, we used *bzIs8* (*P_mec-4_gfp* ), which labels six touch neurons, as marker to monitor the survival of touch neurons and tested the effect of the *eif-3.K* mutation on the missing cell phenotype of the *csp-3(lf)* animals. As previously shown [14], in *csp-3(lf)* animals six touch neurons were lost randomly at a frequency from 2% to 10% (Table S2) and 24% of animals lost at least one touch neuron (Table S2). The *eif-3.K(gk126)* mutation strongly suppressed this missing cell defect in *csp-3* mutants (Table S2). In addition, loss of *csp-2* resulted in increased germline cell deaths [13], and this phenotype can also be suppressed by the *eif-3.K* mutation (Figure S6). These results suggest that EIF-3.K may promote cell death downstream of or in parallel to *csp-2* or *csp-3* in the germline and somatic cell deaths, respectively.

Human eIF3k co-localizes with keratin and requires keratin for its apoptosis-promoting function in simple epithelial cells [30]. Upon apoptotic stimuli, keratin K18 is cleaved by caspase 3 at VEVD^238^ of the L1-2 linker region or DALD^397^ of the C terminal (tail) domain, resulting in a collapse of keratin filaments [67]. *C. elegans* contains eleven genes that encode cytoplasmic intermediate filaments, including *ifa-1*, *mua-6*, *ifa-3*, *ifa-4*, *ifb-1*, *ifb-2*, *ifc-1*, *ifc-2*, *ifd-1*, *ifd-2*, and *ifp-1* [68]. No detectable change in either the localization or the level of the EIF-3.K protein was observed by immunostaining analysis in *mua-6(rh85)* or *ifb-1(ju71)* mutant embryos or in embryos treated with *ifa-1*, *mua-6*, *ifa-4*, *ifb-1*, *ifc-1*, or *ifd-1* interfering RNAs (data not shown). Potential CED-3 cleavage sites (DXXD) are found in IFA-1 (DAED), IFC-2 (DNRD), IFD-1(DNRD and DVDD), and IFP-1 (DSVD). The RNAi-mediated inactivation of *ifa-1*, but not *ifc-2*, *ifd-1*, or *ifp-1*, reduced the number of cell corpses at the comma stage. For example, the *ifa-1(RNAi)* worms had on average 6.9±1.8 cell corpses, which is similar to the number of cell corpses observed in *eif-3.K* mutants; however, whether IFA-1, IFC-2, IFD-1, or IFP-1 are direct targets of the CED-3 caspase or are involved in programmed cell death needs to be evaluated. It is not yet clear whether EIF-3.K localizes to intermediate filaments or mediates programmed cell death through intermediate filaments in *C. elegans*, similar to human eIF3k. Previously, the pro-apoptotic function of human eIF3k was identified and assayed in simple epithelial cells [30] in which keratin K8/K18 is the major intermediate filament. It will be interesting to determine whether human eIF3k, like *C. elegans* eIF3.K, can promote apoptosis in muscle or neuron cells, as human eIF3k is widely expressed in many tissues, including the brain and muscle [27], where no or very little keratin is expressed [69]. In addition, because eIF3k is also present in *D. melanogaster*, an organism that lacks intermediate filaments, it will be interesting to see if eIF3k plays a role in apoptosis in *D. melanogaster* as well.

*C. elegans* EIF-3.K and human eIF3k both contain two conserved domains, the WH and HAM domains. The HAM domain, but not the WH domain, of human eIF3k interacts with keratin 18 in a yeast two-hybrid system and thus may be important for eIF3k localization to keratin [30]; however, in *C. elegans*, the HAM domain is dispensable for the cell death-promoting function of EIF-3.K and the WH domain alone is sufficient to promote cell death (Table 3). This result suggests that the WH domain may promote programmed cell death by an IF-independent mechanism. The WH domain has been implicated in DNA or RNA binding [59], but how it may promote programmed cell death needs further study. The result that human eIF3k can partially rescue the cell death defect in the *eif-3.K* mutants suggests that the eIF3k family may promote apoptosis through a conserved mechanism, which may be dependent upon the WH domain.

## Supporting Information

Figure S1 **The identification of extraneous surviving cells in the mutants.** The y axis represents the percentage of animals with specific superfluous surviving cells (x axis). The extra surviving cells are named after their sister or niece cells, such as “e1 sister cell” and “I2 niece cell”. M4, MC, NSM, I1 and I2 are neurons. e1 is an epithelial cell, and m1 and m2 are muscle cells. L: left, R: right. The identities of extraneous surviving cells were determined as previously described [41]. More than 20 worms for each genotype were scored.
(TIF)

Figure S2 **EIF-3.K is not associated with mitochondria.** A wild-type embryo was co-stained with anti-EIF-3.K antibodies (A) and MitoTracker (B). The merged image is shown in C. Scale bar = 10 µm.
(TIF)

Figure S3 **Deletion of the WH domain does not affect the expression pattern or stability of EIF-3.K.** The wild-type embryo (A) and *eif-3.K* mutant embryo (B) with no transgene, and the *eif-3.K* mutant embryos carrying the transgene P*_hsp_eif-3.K* (C), P*_hsp_eif-3. K*ΔHAM (D), or P*_hsp_eif-3. K*ΔWH (E) were heat shocked and co-stained with anti-EIF-3.K antibodies (red) and DAPI (blue). Representative images of anti-EIF-3.K antibody staining (upper panel) and merged images of anti-EIF-3.K antibody and DAPI staining (lower panel) are shown. Scale bar = 10 µm.
(TIF)

Figure S4 **Neither EIF-3.K nor the WH domain alone interacts with CED-3 or CED-4 in a yeast 2-hybrid assay.** Pairs of constructs expressing the indicated fusion proteins were transformed into the yeast strain MaV203. The resulting transformants were streaked on SC-Trp-Leu-His or SC-Trp-Leu plates containing 30 mM 3 AT. Growth on the SC-Trp-Leu-His+30 mM 3 AT plate indicates an interaction between the fusion proteins. The E2F1and RB pair was used as positive control [70]. “-” in the lower panel indicates no insert was present in the AD fusion construct.
(TIF)

Figure S5 **Loss of** ***eif-3.K*** **reduced DNA damage-induced apoptosis.** Apoptotic germ cell corpses were scored in the wild-type (black columns) and *eif-3.K(gk126)* (white columns) young adult worms 24 hr following exposure to 150 J/m^2^ UV-C radiation. The *eif-3.K(gk126)* mutants were compared to the wild-type using the unpaired t test (**P<0.001). More than 20 gonadal arms were scored for each genotype.
(TIF)

Figure S6 **Loss of** ***eif-3.K*** **suppressed the increased cell death phenotype of** ***csp-2*** **mutants in the germline.** Germ cell corpses were scored in *ced-6(n2095)* (white columns), *ced-6(n2095); csp-2(tm3077)* (gray columns), *ced-6(n2095); eif-3.K(gk126)* (black columns), *ced-6(n2095); csp-2(tm3077); eif-3.K(gk126)* (slashed columns) worms 48 hours after entering adulthood. The y axis represents the average number of cell corpses scored in each gonadal arm. The data were compared using the unpaired t test (*P<0.05, **P<0.001). More than 20 gonadal arms of each genotype were scored.
(TIF)

Table S1 ***eif-3.K*** **is not essential for embryonic or larval development.**
(DOC)

Table S2 **The missing cell defect in** ***csp-3*** **mutants was suppressed by loss of** ***eif-3.K*** **.**
(DOC)
