# Supplementary figures and images for: C. elegans EIF-3.K Promotes Programmed Cell Death through CED-3 Caspase
Source: PLoS One. 2012 May 9;7(5):e36584. doi: 10.1371/journal.pone.0036584 (PMC3348885; doi:10.1371/journal.pone.0036584)

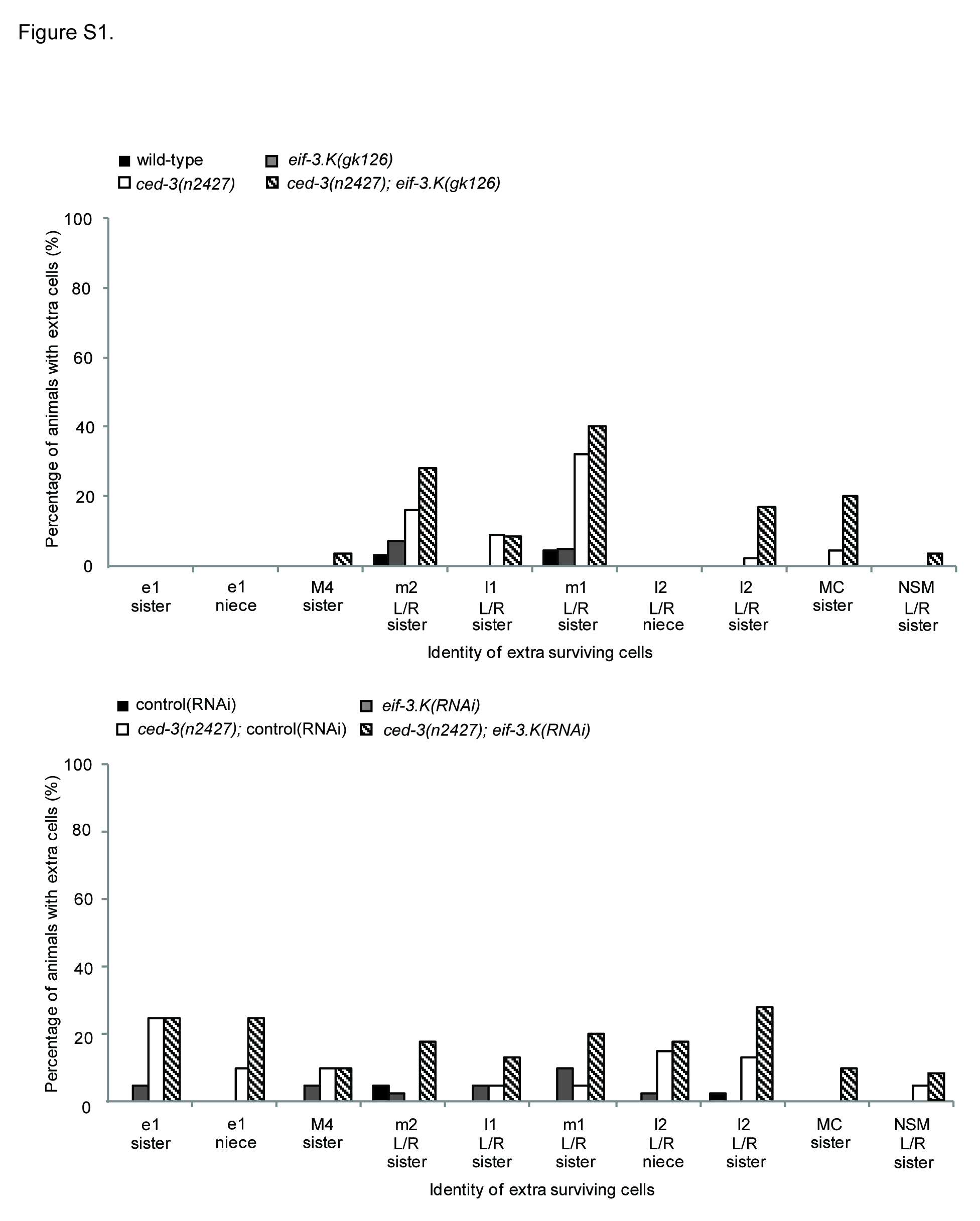

Supplement: Figure S1 — The identification of extraneous surviving cells in the mutants. The y axis represents the percentage of animals with specific superfluous surviving cells (x axis). The extra surviving cells are named after their sister or niece cells, such as “e1 sister cell” and “I2 niece cell”. M4, MC, NSM, I1 and I2 are neurons. e1 is an epithelial cell, and m1 and m2 are muscle cells. L: left, R: right. The identities of extraneous surviving cells were determined as previously described [41]. More than 20 worms for each genotype were scored. (TIF) [file pone.0036584.s001.tif]

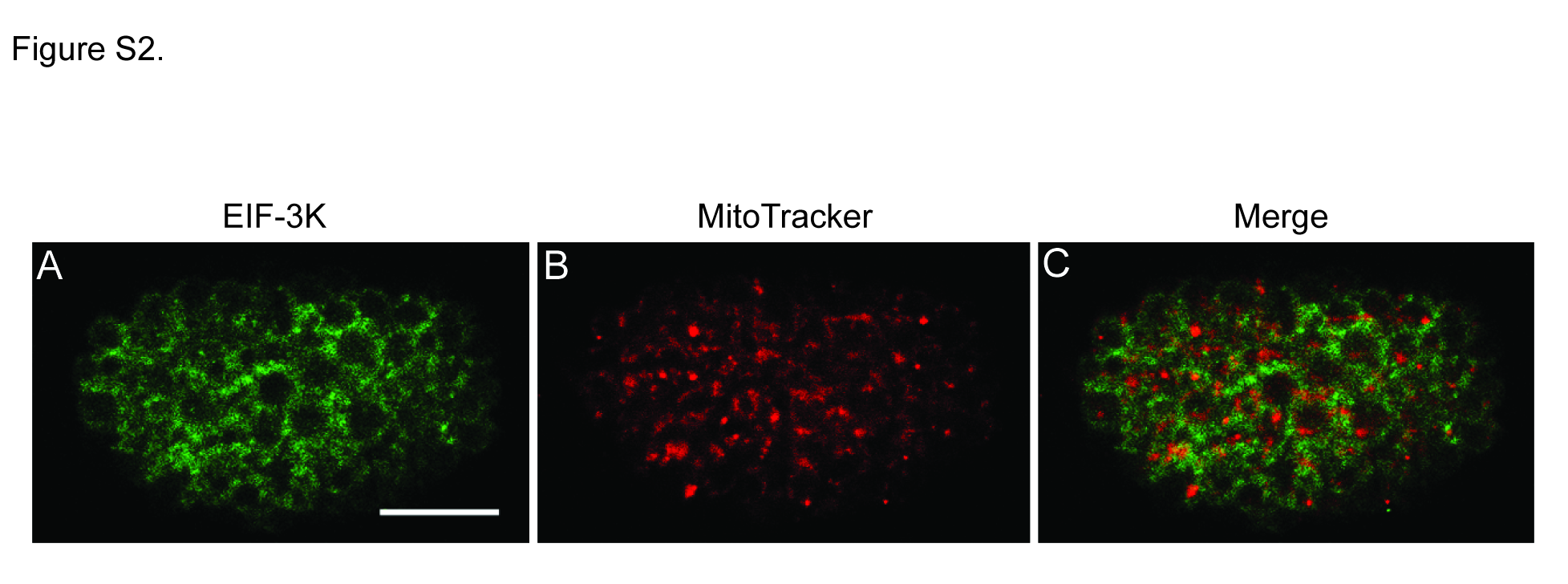

Supplement: Figure S2 — EIF-3.K is not associated with mitochondria. A wild-type embryo was co-stained with anti-EIF-3.K antibodies (A) and MitoTracker (B). The merged image is shown in C. Scale bar = 10 µm. (TIF) [file pone.0036584.s002.tif]

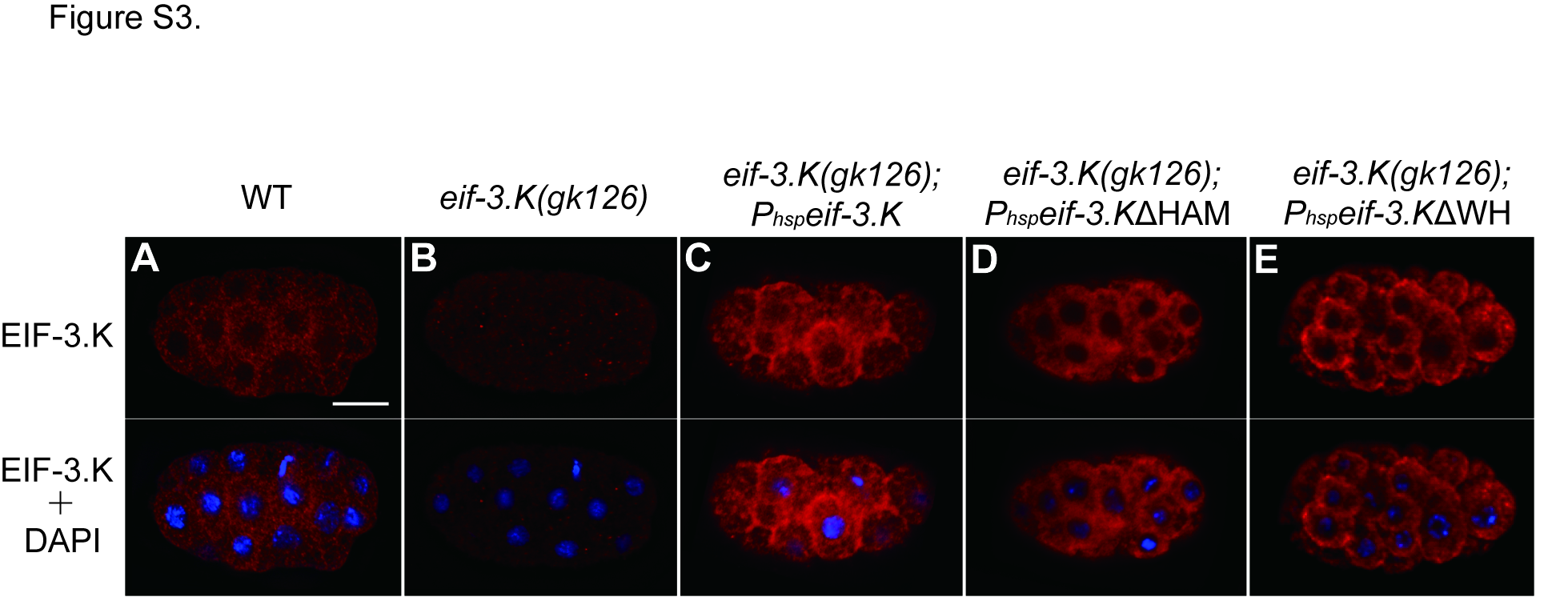

Supplement: Figure S3 — Deletion of the WH domain does not affect the expression pattern or stability of EIF-3.K. The wild-type embryo (A) and eif-3.K mutant embryo (B) with no transgene, and the eif-3.K mutant embryos carrying the transgene Phspeif-3.K (C), Phspeif-3. KΔHAM (D), or Phspeif-3. KΔWH (E) were heat shocked and co-stained with anti-EIF-3.K antibodies (red) and DAPI (blue). Representative images of anti-EIF-3.K antibody staining (upper panel) and merged images of anti-EIF-3.K antibody and DAPI staining (lower panel) are shown. Scale bar = 10 µm. (TIF) [file pone.0036584.s003.tif]

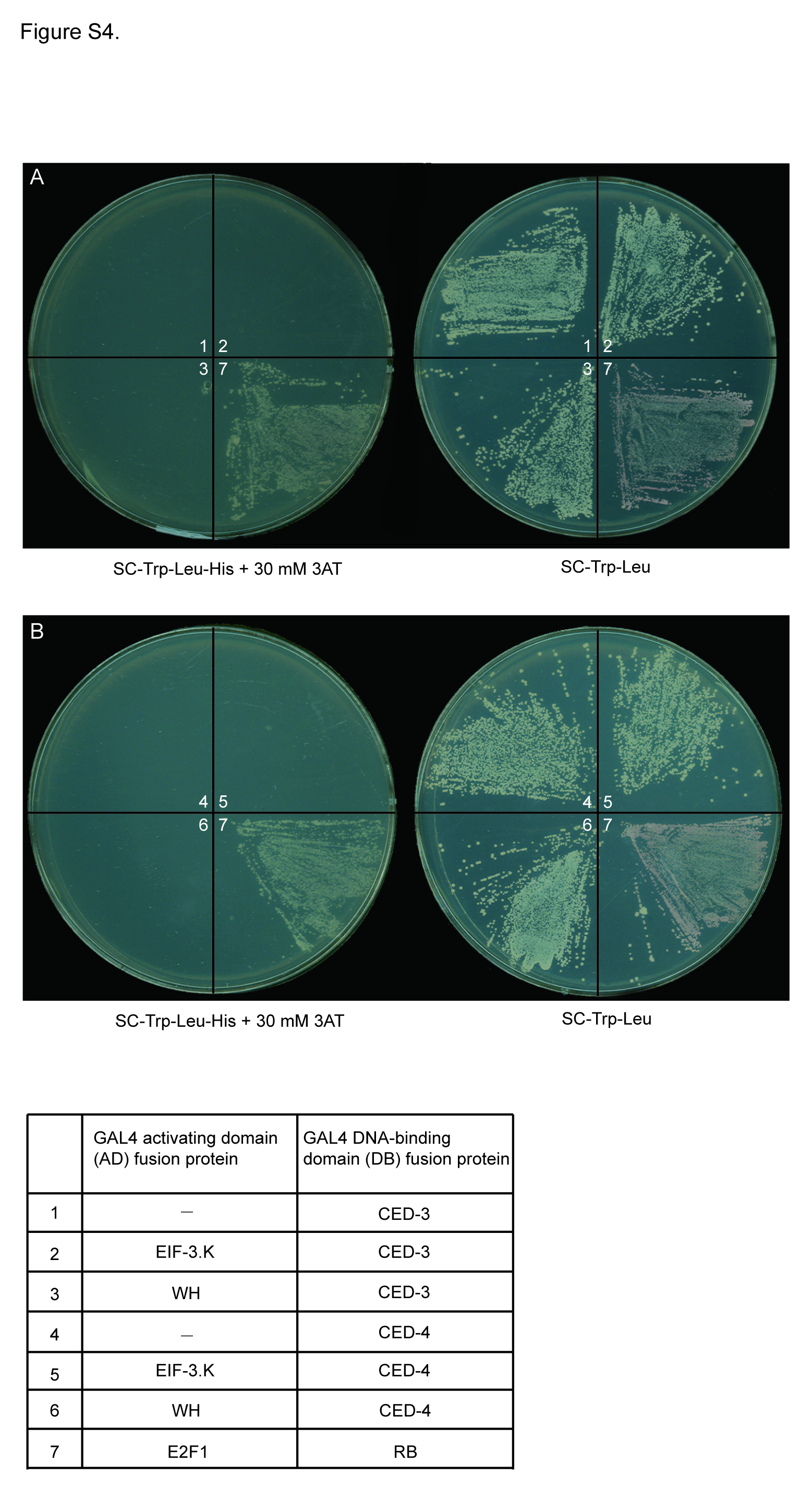

Supplement: Figure S4 — Neither EIF-3.K nor the WH domain alone interacts with CED-3 or CED-4 in a yeast 2-hybrid assay. Pairs of constructs expressing the indicated fusion proteins were transformed into the yeast strain MaV203. The resulting transformants were streaked on SC-Trp-Leu-His or SC-Trp-Leu plates containing 30 mM 3 AT. Growth on the SC-Trp-Leu-His+30 mM 3 AT plate indicates an interaction between the fusion proteins. The E2F1and RB pair was used as positive control [70]. “-” in the lower panel indicates no insert was present in the AD fusion construct. (TIF) [file pone.0036584.s004.tif]

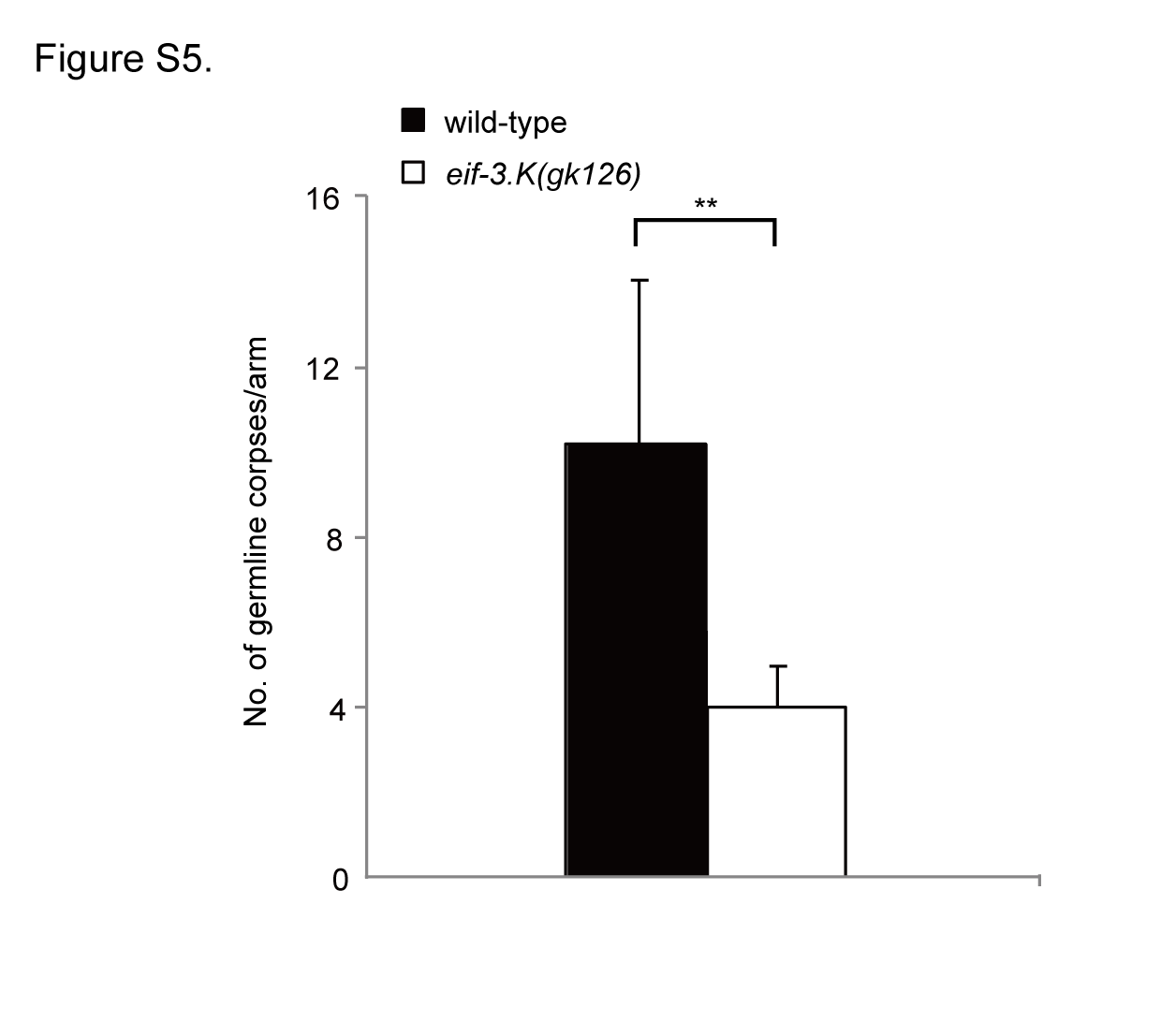

Supplement: Figure S5 — Loss of eif-3.K reduced DNA damage-induced apoptosis. Apoptotic germ cell corpses were scored in the wild-type (black columns) and eif-3.K(gk126) (white columns) young adult worms 24 hr following exposure to 150 J/m2 UV-C radiation. The eif-3.K(gk126) mutants were compared to the wild-type using the unpaired t test (**P<0.001). More than 20 gonadal arms were scored for each genotype. (TIF) [file pone.0036584.s005.tif]

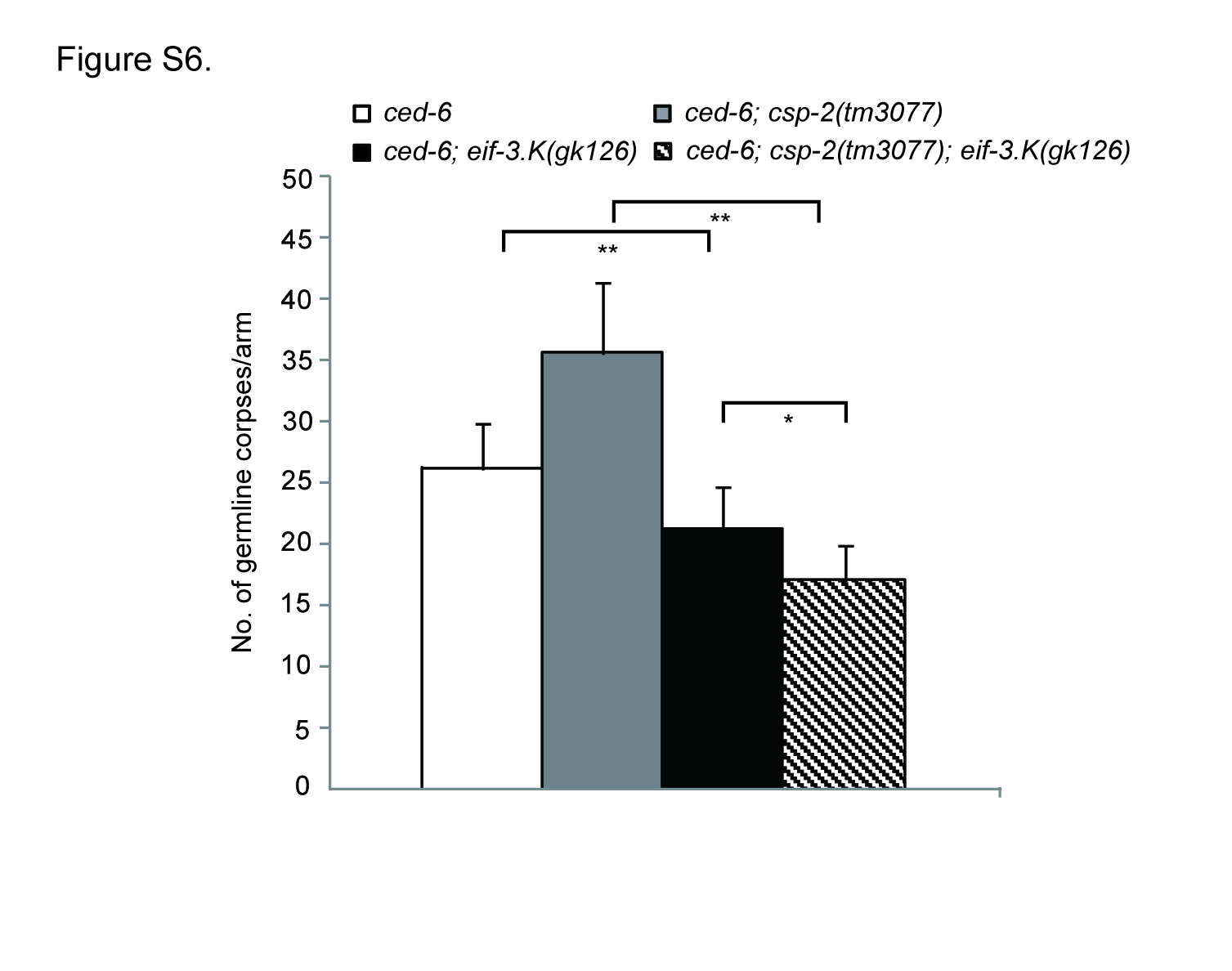

Supplement: Figure S6 — Loss of eif-3.K suppressed the increased cell death phenotype of csp-2 mutants in the germline. Germ cell corpses were scored in ced-6(n2095) (white columns), ced-6(n2095); csp-2(tm3077) (gray columns), ced-6(n2095); eif-3.K(gk126) (black columns), ced-6(n2095); csp-2(tm3077); eif-3.K(gk126) (slashed columns) worms 48 hours after entering adulthood. The y axis represents the average number of cell corpses scored in each gonadal arm. The data were compared using the unpaired t test (*P<0.05, **P<0.001). More than 20 gonadal arms of each genotype were scored. (TIF) [file pone.0036584.s006.tif]
