# Supplementary material for: C. elegans EIF-3.K Promotes Programmed Cell Death through CED-3 Caspase
Source: PLoS One. 2012 May 9;7(5):e36584. doi: 10.1371/journal.pone.0036584 (PMC3348885; doi:10.1371/journal.pone.0036584)
Supplement: Table S1 — eif-3.K is not essential for embryonic or larval development. (DOC) [file pone.0036584.s007.doc]

| Table S1. *eif-3.K* is not essential for embryonic or larval development. | | |
| --- | --- | --- |
| Genotype | Embryonic lethality^a^ (%) | Embryos reaching adulthood in 3 days after hatching^b^ (%) |
| wild-type | 1.2 | 97.2 |
| *eif-3.K(gk126)* | 1.4 | 100.0 |
| ^a^ To assay embryonic lethality, total embryos laid by young adults in the first 3 days were scored and the embryos that fail to hatch within 24 hrs were counted as lethal. Greater than 1000 embryos for each genotype were analyzed.  ^b^ The percentage of embryos that reach adulthood in three days after hatching were calculated. Greater than 1000 embryos for each genotype were analyzed. | | |
