# Supplementary material for: C. elegans EIF-3.K Promotes Programmed Cell Death through CED-3 Caspase
Source: PLoS One. 2012 May 9;7(5):e36584. doi: 10.1371/journal.pone.0036584 (PMC3348885; doi:10.1371/journal.pone.0036584)
Supplement: Table S2 — The missing cell defect in csp-3 mutants was suppressed by loss of eif-3.K . (DOC) [file pone.0036584.s008.doc]

| Table S2. The missing cell defect in *csp-3* mutants was suppressed by loss of *eif-3.K*. | | | | | |
| --- | --- | --- | --- | --- | --- |
| Genotype | Cell missing(%)^a^ | | | | Animals missing at least one neuron(%)^b^ |
|  | AVM | ALMR/L | PVM | PLMR/L |  |
| *bzIs8* | 0% | 0.5% | 0% | 0% | 1% |
| *csp-3(tm2486);bzIs8* | 5% | 9% | 7% | 2% | 24% |
| *csp-3(tm2486);bzIs8;eif-3.K(gk126)* | 0% | 0.5% | 0% | 0% | 1% |
| ^a^The percentage of the indicated touch neuron lost was shown.  ^b^The percentage of animals missing at least one touch neuron is shown. An integrated transgene (*bzIs8*) was used to monitor the survival of six touch neurons as described in Methods. At least 100 animals were scored for each genotype. | | | | | |
